# Structural Health Monitoring of Glass Fiber-Reinforced Polymer Laminates with Carbon Nanotube-Coated Glass Fiber Sensing Layer after Low-Velocity Impact Using Electrical Resistance Tomography

**DOI:** 10.3390/nano14171462

**Published:** 2024-09-09

**Authors:** Zijie Zhao, Minglong Li, Ya Liu, Anhua Wang, Biaojun Zhou, Junfeng Hu

**Affiliations:** 1 National Key Laboratory of Transient Physics, Nanjing University of Science and Technology, Nanjing 210094, China; wah1996@njust.edu.cn (A.W.); zhoubiaojun@njust.edu.cn (B.Z.); 2 School of Mechanical and Power Engineering, Nanjing Tech University, Nanjing 211800, China; 202261107045@njtech.edu.cn (M.L.); 202361207198@njtech.edu.cn (Y.L.); junfeng-hu@njtech.edu.cn (J.H.); 3 Jiangsu Olymspan Thermal Energy Equipment Co., Ltd., Changzhou 213101, China; 4 Suzhou Yihe Yongli New Energy Co., Ltd., Suzhou 215400, China

**Keywords:** carbon nanotubes, sensing fibers, damage monitoring, ERT imaging, low-velocity impact

## Abstract

Structural health monitoring (SHM) of composite materials is of great significance in various practical applications. However, it is a challenge to accurately monitor the damage of composites without affecting their mechanical properties. In this paper, an embedded sensing layer based on carbon nanotube-coated glass fiber is designed, combined with electrical resistance tomography (ERT) for in situ damage monitoring. Multi-wall carbon nanotube-coated glass fiber (MWCNT-GF) is prepared and embedded into laminates as an in situ sensing layer. Low-velocity impact experiments demonstrate that the embedded sensing layer has high compatibility with the composite laminates and has no adverse effect on its impact response; although, the energy absorption behavior of glass fiber-reinforced polymer (GFRP) laminates containing MWCNT-GF occurs about 10% earlier than that of GFRP laminates overall. ERT technology is used to analyze the laminates after a low-velocity impact test. The results show that the in situ monitoring method with the embedded MWCNT-GF sensing layer can achieve high precision in imaging localization of impact damage, and the error of the detected damage area is only 4.5%.

## 1. Introduction

Fiber-reinforced polymer composites (FRP) are widely used in aerospace, civil engineering, rail transit, and other fields due to their high specific strength, specific modulus, and fatigue resistance [1,2,3]. However, the composite materials will inevitably encounter low-velocity impact (LVI) during service, which could cause matrix cracks, fiber debonding, delamination, and other imperceptible internal damages between the reinforcement phase and the matrix [4,5]. These internal damages will lead to the catastrophic failure of the materials without warning after long-term accumulation and propagation [6,7]. Therefore, damage monitoring and evaluation of composite materials are particularly important. The present mainstream non-destructive testing (NDT) technologies for damage detection, including optical fiber, X-ray monitoring, infrared monitoring, and acoustic emission, are considered reliable, but the limitations of off-ground and off-line damage monitoring technologies have not been solved [8,9]. These problems inevitably result in increased downtime for composite structures and maintenance costs.

In order to overcome these limitations, a structural health monitoring method based on electrical sensors was proposed which could continuously track the internal state of composite materials during use and realize real-time damage monitoring [10,11,12]. Moreover, such sensors can not only be installed externally, but also integrated inside the composite material. External sensors usually have no effect on the mechanical properties of composite structures, but long-term exposure to the external environment may lead to reduced accuracy of sensors. In contrast, internal sensors provide a high signal-to-noise ratio, stability, and durability. In addition, a sensor material similar to the main composite material is used for the internal integrated sensor, which results in little influence on the mechanical properties of the composite structures [13,14].

In recent years, carbon nanomaterials have been widely used in the field of structural sensing detection because of their excellent mechanical and electrical properties. Under quasi-static loading, carbon nanotubes (CNTs) [15,16], graphene nanosheets (GnPs) [17,18], resin films [19], etc., have significant effects capable of improving the interlaminar strength of the composites. Among them, double-walled carbon nanotubes (MWCNTs) are one of the most functional and economically competitive modified materials. By adding MWCNTs to the matrix, the mechanical and electrical properties of composites could be enhanced significantly [20,21]. Gojny et al. [22] showed that by adding only 0.1% MWCNTs to epoxy resin, the fracture toughness of composites could be increased by 17%. Kim [21] added 3.0 wt.% nano-clay to epoxy resin via the three-roll milling method, achieving a 50% improvement in fracture toughness, demonstrating the differentiated effects of different nano-fillers on the properties of epoxy resin. In addition, the carbon nanofiber interlayer prepared by Arai [23] using steam growth technology significantly enhanced the fracture toughness of FRP laminates. Zheng Zhicai [24] prepared a surface modifier containing MWCNTs using the ultrasonic method and applied it to the surface of carbon fiber. The research showed that the tensile strength of the modified carbon fiber increased by 59.4%.

The application of carbon nanotubes as functional fillers has also attracted wide attention, especially in the aspects of health monitoring and damage sensing of FRPs. This method uses the formation of conductive channels to identify different damage modes of composites under load by measuring and analyzing electrical signals [25,26]. Park [27] combined the change in resistance of carbon nanotubes with acoustic emission technology to explore the effect of CNT content on the change in the resistance of composites. Low levels of CNTs (0.1 wt%) lead to a sharp increase in resistance when the carbon fiber fractures, indicating that CNTs do not form a stable conductive network in the composite. In addition, Reghat et al. [28] provided a new method for real-time damage monitoring by applying graphene to the surface of glass fiber fabric and preparing composite materials using the vacuum resin impregnation method. Dhiwar et al. [29] proposed a strategy to simultaneously enhance fracture toughness and the damage sensing of Kevlar composites by using carbon nanotubes. Wang [9] introduced carbon nanoparticles/fibers (CNFs) with a high length-to-diameter ratio into epoxy resin through mechanical mixing and ultrasonic dispersion to study their piezoresistance response under different load conditions. The experimental results showed that CNFs/epoxy composites exhibited stable and repeatable piezoresistive properties under either monotone or cyclic load conditions. Yin [30] realized real-time deformation monitoring of composite materials by forming a conductive network of carbon nanotubes on the surface of aramid fibers. Bregar Tadej [31] successfully monitored the strain change in the aluminum bond joint by adding carbon nanotubes to the high-strength epoxy adhesive, accurately indicating the bonding failure under different loads.

Compared with the use of resistance change to determine whether the structure has been damaged, ERT technology is more accurate and effective, which is an imaging technique based on differences in the electrical conductivity of internal materials of structures. By applying an exciting current or voltage to the surface of the structure, the ERT technique can measure and map the distribution of internal electrical conductivity, thereby reconstructing the image of the structure’s internal resistance. For example, Ali Zarafshani et al. [32] demonstrated the potential of this technology in layer separation and damage detection of CFRP composites by designing planar ERT sensor arrays. Wenru Fan et al. [33] proposed an electrical impedance tomography method based on 6 × 6 electrode arrays and found that four excitation strategies had the best performance on the interval modes and were suitable for anisotropic CFRP damage detection, by comparing with five excitation strategies. However, Baltopoulos et al. [34] measured the change in the potential field using ERT technology and provided the conductivity change map to identify the damage in the carbon nanotube network. The damage sensitivity was less than 0.1% of the detection area, and the positioning monitoring error was close to 10%. These studies show that ERT technology has significant advantages in CFRP structural health monitoring.

The above research provides the possibility for damage monitoring of fiber-reinforced composite laminates with an embedded sensing layer under quasi-static load. Since the bending properties of composite laminates are most easily affected by heterogeneous layers, these embedded sensing layers may have a negative effect on the impact resistance of composite laminates under dynamic impact loads. In addition, the sensitivity of sensor data acquisition is critical for accurately locating damage areas in composites. In this paper, MWCNTs are used to modify the surface of glass fiber, and the modified fiber is embedded in the middle of the laminate in the form of an array. Since ERT analysis is usually applied to planar membrane structures, in this study, ERT imaging detection is realized by equating the fiber array to a continuous membrane layer. Although the fiber array is discrete in structure, it can be regarded as a continuous conductive film in terms of electrical property. By measuring the resistance of these electrode pairs, the detected resistance change is regarded as the response of the whole film layer, and ERT imaging technology is applied to reconstruct the resistance distribution of the fiber array before and after impact. Although the fiber array structure may not be completely uniform in practical applications, the normalization of the initial resistance data ensures that the resistance image before impact is evenly distributed during ERT imaging. The resistance data between electrodes of the laminate after impact are processed accordingly to make them change based on the initial normalized data to ensure that ERT imaging results accurately reflect the damage caused by impact. Based on this method, ERT imaging technology can be used more accurately to monitor the structural health of composite laminates embedded with MWCNT-GF sensors, which provides an effective technical way for health monitoring and damage warning of composite structures.

## 2. Experimental Method

### 2.1. Materials

The MWCNTs used in this study were purchased from Shanghai McLean Reagent Co., Ltd. (Shanghai, China). with an average diameter and length of 30–50 nm and 10–20 μm, respectively. Sodium dodecyl benzene sulfonate (SDBS) was selected as the dispersant, which was provided by Wuxi Yatai United Chemical Co., Ltd. (Wuxi, China). Continuous glass fiber unidirectional prepregs and the direct roving (600 TEX) of the same glass fiber were provided by Weihai Guangwei Group Co., Ltd. (Weihai, China). for fabricating GFRP laminates and MWCNT-GF. Acetone, ethanol, and deionized water were purchased from Sinopharm Group Chemical reagent Co., Ltd. (Shanghai, China).

### 2.2. Preparation of MWCNT-Modified Glass Fiber

**Figure 1:**
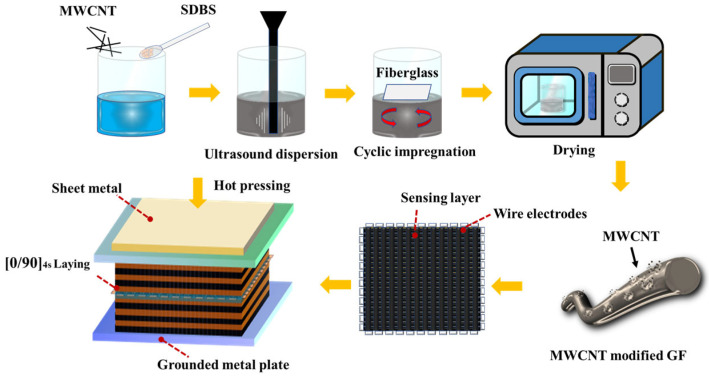
Preparation process of MWCNT-modified fiber sensing layer.

In this study, the MWCNT-modified sensing glass fiber was prepared via the liquid phase solvent deposition method. Due to the high specific surface area and strong van der Waals forces of carbon nanotubes, their tendency to easily aggregate in solution may lead to a dispersion problem, further affecting their uniform distribution on glass fibers and related properties. SDBS contains hydrophilic groups, which can form hydrogen bonds with water molecules, and hydrophobic groups, which bind to the surface of carbon nanotubes through hydrophobic interaction. This results in the formation of a stable complex of SDBS–MWCNTs. Due to the electrostatic repulsion of hydrophilic groups, the reaggregation of carbon nanotubes is inhibited, and the stable dispersion of carbon nanotubes in solution becomes possible [35,36]. Therefore, SDBS is used as a dispersant to improve the dispersion ability of carbon nanotubes in this study. In addition, the conductivity of MWCNTs in the sensing layer is largely dependent on the integrity of the formed conductive network. A variety of concentration matching experiments are designed, as given in Table 1. The concentration of carbon nanotubes (N) in deionized water is set to 3.75, 5, 6.25, and 7.5 mg/mL, respectively. The concentration ratio of dispersant to carbon nanotubes (M) is set to 0.26, 0.3, and 0.34. The carbon nanotube solution is obtained via mechanical stirring at 30 °C and 500 rpm for 90 min, followed by ultrasonic treatment at 60 W of power for 3 h. On the other hand, the effect of impregnation number on the conductivity of WCNT-GF is analyzed. Before the impregnating of MWCNTs, the glass fiber bundles are cleaned with alcohol to remove any dust and then are impregnated in MWCNT solution for 5 min each time. Finally, the impregnated glass fibers are dried in an oven at 80 °C for one hour to obtain MWCNT-modified glass fibers, as shown in Figure 1. A data collector (Keithley DAQ6510, Tektronix Technology Co., Ltd., China) is used to measure the resistance of MWCNT-GF, and the optimal impregnation scheme is determined by comparing the resistance values. A scanning electron microscope (JSM-7600F, Nippon Electronics Co., Ltd., Japan) is used to scan the microstructure of the fiber surface, which further confirms the reliability of the experimental results.

### 2.3. Fabrication of GFRP Laminates with MWCNT-GF

**Figure 2:**
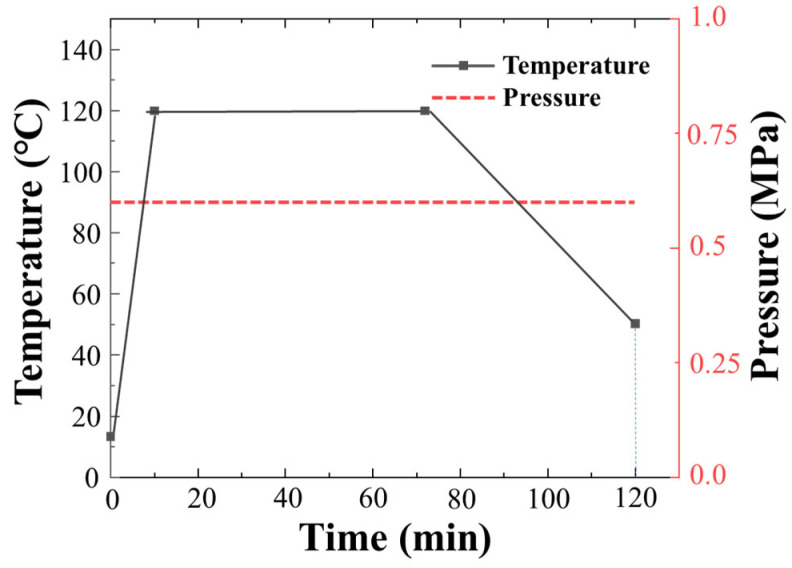
Curing curve of GFRP laminates with and without sensing layer.

The glass fiber prepreg is cut to the designed size of 100 × 100 mm using a CNC cutting machine and then laid according to the orthogonal stacking sequence of [0/90]_4s_. Then, carbon nanotube-modified glass fibers are embedded in the middle layer as the sensing layer, and 9 bundles of glass fiber are inserted horizontally and vertically, respectively, to ensure the integrity of the conductive path. The ultra-thin fine copper film with a thickness of 0.1 mm is used as the electrode to lap on the glass fiber, and there are 4 electrodes on each side for a total of 16 electrodes. Finally, the prepregs with and without the sensing layer are hot pressed on a molding machine with a pressure of 0.6 MPa, a temperature of 120 °C for 60 min, and a heating rate of about 12 °C/min, following the curing cycle shown in the Figure 2. After cooling to room temperature, GFRP laminates with a thickness of 2.2 mm are obtained. In this research, there are 4 specimens with and without sensing fibers, respectively, which were cut from the corresponding same laminate to ensure the accuracy of experimental data.

### 2.4. Low-Velocity Impact Test

**Figure 3:**
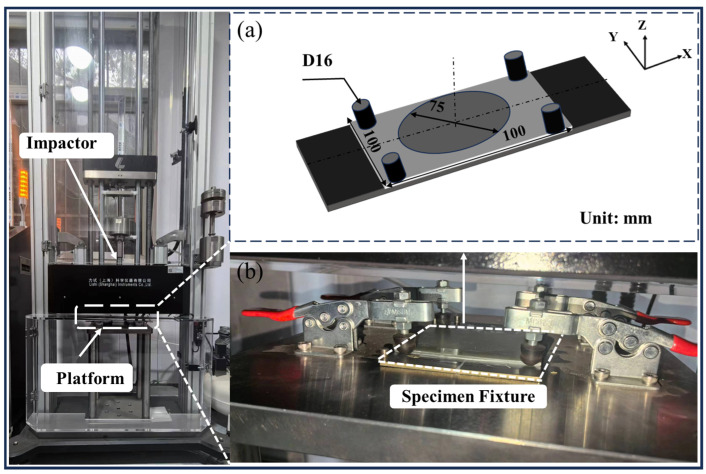
Drop weight impact testing machine and the experimental setup: (**a**) the support fixture system, and (**b**) specimen installation.

A low-velocity impact test is carried out on composite laminates with and without the embedded sensing layer. In this study, fabricated laminates are typical thin-walled structures with a thickness of about 2.2 mm, and the impact energy is selected as 20 J according to the previous study on the low-velocity impact behavior of composite laminates [5]. Figure 3 shows the low-velocity impact experiment setup using a drop weight impact testing machine (LI5000, Lishi Instruments Co., Ltd., Shanghai, China). The specimen is fixed between two steel plates with four pressure clamps, leaving a 75 mm diameter impact area in the middle. According to the low-velocity impact test standard ASTM D7136, the weight of the drop hammer is 5.265 kg, the head of the drop hammer is 16 mm in diameter, and the punch axis is perpendicular to the plane of the laminate.

### 2.5. ERT Monitoring Technology

Based on the continuity of current and Ohm’s law, to establish accurate mathematical models and algorithms, ERT technology can accurately detect the resistivity changes inside the composites and can be used to monitor the internal damage of the structures under load or environmental changes. Given a domain Ω, which is the two-dimensional analysis region in the present research, the resistivity distribution is ρ(x) when the current is injected through an electrode. The variable *x* represents the location point in the analysis region. The potential u(x) satisfies the following partial differential equation according to Ohm’s law and the law of conservation of charge.

(1) ∇·(σ(x)∇u(x))=0,x∈Ω

where σ(x)=1/ρ(x) is the electrical conductivity. At the boundary ∂Ω, the corresponding current and potential conditions can be applied depending on the electrode and boundary conditions.

The positive problem of ERT, that is, given the resistivity distribution ρ(x), calculates the potential distribution u(x) in the field of Ω. This is a boundary value problem and can usually be solved with numerical methods, such as the finite element method and boundary element method, etc. The inverse problem of ERT is to invert the internal resistivity distribution ρ(x) from the measured boundary potential data. The present image reconstruction algorithms used to solve ERT inverse problems are mostly based on sensitivity matrix J, which also becomes an important part of ERT positive problems to simulate the effect of resistivity distribution change on potential distribution under a small disturbance, and its linearized physical model could be expressed as follows:

(2) Jδσ=δU

where δσ is the change value of conductivity, δU is the corresponding change value of boundary measurement voltage, and the sensitivity matrix J is based on the change in boundary measurement voltage with a small change in conductivity in the field, thereby linearizing the ERT problem. For the electrode excitation system, the adjacent electrode model and the complete electrode model (CEM) are commonly used. The adjacent electrode model refers to a configuration in which current is injected and measured between adjacent pairs of electrodes. As for the CEM model, it takes into account the influence of the contact impedance of each electrode, giving a more complete representation of the boundary conditions. According to the CEM model, the boundary conditions of Equation (2) could be expressed as follows:

(3) $$u+z_{l}\sigma \frac{\partial u}{\partial n}=U_{l},l=1,2\ldots L$$

(4) $$\int_{el}\sigma \frac{\partial u}{\partial n}dS=I_{l},l=1,2\ldots L$$

(5) $$\sigma \frac{\partial u}{\partial n}=0$$

(6) $$\sum_{l=1}^{L}I_{l}=0$$

(7) $$\sum_{l=1}^{L}U_{l}=0$$

where $z_{l}$ is the effective contact impedance between the electrode and the sensitive medium. *I_l_* and $U_{l}$ are the current and voltage on the electrode $e_{l}$. n is the outgoing unit normal vector. *u* is the electric potential. L is the number of electrodes. In particular, Equations (3)–(5) define the sum of the current and voltage of the edge electrodes and other regions, taking into account the shunt effect of each electrode and the contact impedance. In addition, Equations (6) and (7) ensure the existence and uniqueness of the solution.

In the positive problem, assuming that the internal distribution of the conductivity within the sensitive medium is known, the aim of ERT analysis is to determine the distribution of the conductivity based on the injection current and the measured boundary voltage. The ERT inverse problem is the process of solving the change in conductivity distribution in the sensitive medium after some events and reconstructing the image. To solve this problem, regularization techniques such as Tikhonov regularization [37] and G_N regularization [38] are usually required to stabilize the solution process. In this study, the voltage variation between electrodes of composite laminates before and after impact is recorded with a data collector using the all-electrode measurement method, which is an integrated method in ERT technology. All possible electrode pairs are used for current injection and voltage measurement, which ensures that the dataset includes the complete set of measurements, thus maximizing the use of information that could be used to reconstruct the conductivity distribution inside the material. Electrical impedance tomography is performed using MATLAB and the open source EIDORS package to obtain more accurate internal imaging results, which could solve the positive and inverse problems, estimating the internal resistivity distribution by solving the sensitivity matrix J. During the solving process, the finite element method is used to divide the imaging region and discretize it into multiple grid cells, each corresponding to a specific resistivity value. The EIDORS package provides the tools to implement these complex calculations, making the imaging results more accurate. In order to eliminate the effect of the resistance non-uniformity between the initial electrodes on the imaging results, a normalization method is adopted in this study to process the resistance data into a uniform film. It is because the difference of initial resistance between the electrodes leads to the uneven voltage distribution that the accuracy of the resistivity image is affected. Standardization can balance out these differences, resulting in more uniform and precise imaging results. Consider the electrode array as a two-dimensional grid, with each grid cell representing a region of one electrode, and assume that the electrode array is a 4 × 4 rectangular array with a total of 16 electrodes and a distance of d = 15 mm between each electrode. The measured resistance data $R_{i}$ (i = 1 to 120) are reconstructed into a 4 × 4 matrix $R_{ij}$, where $R_{ij}$ represents the resistance value of the electrodes in row i and column j. For each grid cell, the weighted average of its resistivity is calculated to process the normalization effect in the imaging process. Assuming that the area of each electrode is $A=d^{2}$, the resistivity $R_{uniform}$ of the homogeneous film could be calculated by the following formula. This step improves the image quality by normalizing the measured resistance value to reduce imaging errors caused by resistance inhomogeneity, taking $R_{uniform}$ as a standard value.

(8) $$R_{uniform}=\frac{1}{A}\sum_{i=1}^{4}\sum_{j=1}^{4}R_{ij}.A$$

## 3. Results and Discussion

### 3.1. Analysis of Conductivity and Surface Morphology of MWCNT-GF

**Figure 4:**
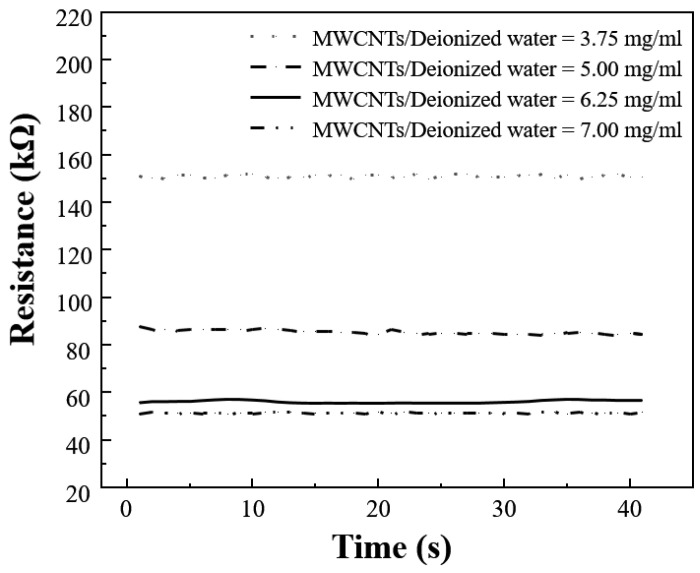
The change in MWCNT-GF resistance after impregnation in solutions with different concentrations of MWCNTs.

**Figure 5:**
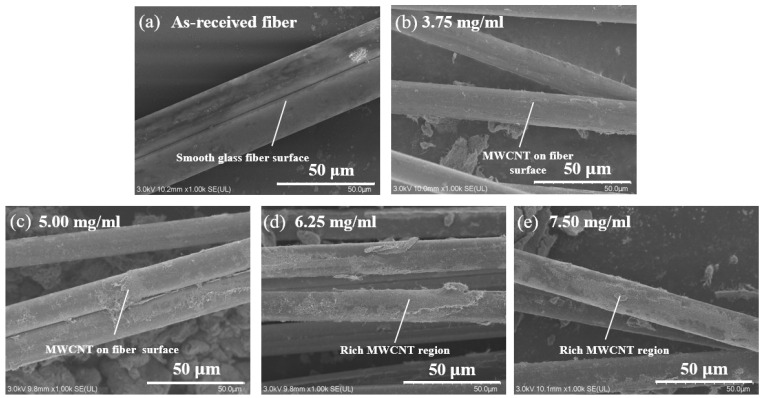
SEM micrographs of MWCNT-GF after impregnation in solutions with different concentrations of carbon nanotubes: (**a**) surface of as-received fibers, (**b**) 3.75 mg/mL, (**c**) 5.00 mg/mL, (**d**) 6.25 mg/mL, and (**e**) 7.50 mg/mL.

The sensing capability of the MWCNT-GF sensing layer depends to a large extent on the perfection of the formed conductive network of MWCNTs. Since the MWCNTs must be sufficient to maintain the appropriate morphology and length-diameter ratio in the conductive network, the MWCNT solution concentration plays a key role in the formation of the MWCNT conductive network. Meanwhile, the dispersant SDBS also has a significant effect on the dispersibility of carbon nanotubes. To prepare MWCNT-GF with good conductivity, it is necessary to optimize the concentration of carbon nanotubes and its ratio with dispersant. It can be seen from Figure 4 that with the increase in MWCNT concentration, the resistance value of MWCNT-coated glass fiber gradually decreases, and when the MWCNT concentration is N = 6.25 and 7.50 mg/mL, the resistance value of modified glass fiber is quite close, indicating that the MWCNT conductive network tends to be saturated in this concentration range. It could be inferred that when the concentration of carbon nanotubes reaches 6.25 mg/mL, the MWCNTs form a sufficiently dense conductive network on the surface of the glass fiber, and the effect of further increasing the concentration on the resistance reduction is no longer significant. Figure 5 shows scanning electron microscope (SEM) images of glass fibers impregnated in MWCNT solutions of different MWCNT concentrations. With the increase in MWCNTs concentration, the MWCNTs coated on the glass fiber surface gradually increase and become more uniform. At the concentration of 5.00 mg/mL, the MWCNTs are relatively thin on the fiber surface and no continuous conductive network has been formed. For the concentrations of 6.25 and 7.50 mg/mL, more MWCNTs appeared on the fiber surface, and the MWCNTs’ distribution gradually became more abundant and uniform. When the carbon nanotube concentration reaches 6.25 mg/mL, the resistance change is very limited even if the carbon nanotube concentration is increased, indicating that the MWCNT network structure has reached the permeability threshold of the system and can form a stable and effective conductive network on the surface of glass fibers. Therefore, choosing N = 6.25 mg/mL as the optimal concentration for the preparation of the MWCNT-based sensing layer not only ensures the formation of a stable and efficient conductive network but also obtains the best balance between use efficiency of material and cost.

**Figure 6:**
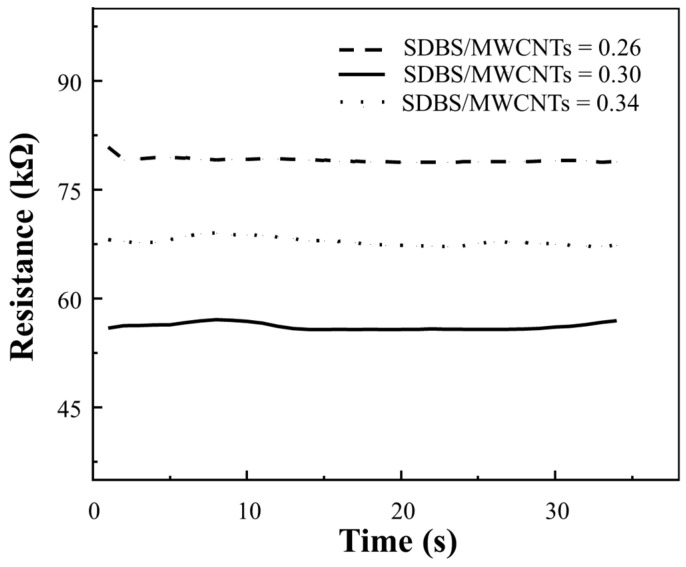
The change in MWCNT-GF resistance after impregnation in solution with different dispersant concentrations.

The mass ratio of dispersant also affects the permeability threshold of MWCNTs. Figure 6 shows the resistance change in glass fibers after impregnation at different SDBS/MWCNT ratios. As the proportion of SDBS/MWCNTs increased from 0.26 to 0.34, the resistance value showed a trend of decreasing at first and then increasing. This phenomenon demonstrates that at the right concentration of SDBS, the dispersion of carbon nanotubes is improved, thus forming a more continuous conductive network. However, when the concentration of SDBS is too high, excessive SDBS may lead to the weakening of the force between carbon nanotubes and affect the formation of a conductive network. The resistance value is the lowest at the SDBS/MWCNT ratio of 0.3, indicating the best dispersion and forming the most effective conductive network at this ratio. The charge shielding effect plays a key role in this process, and the hydrophilic head group in the molecular structure of SDBS has a negative charge. When the concentration of SDBS is moderate, these negative charges help to prevent the aggregation of carbon nanotubes via electrostatic repulsion, thereby improving the dispersion. However, when the concentration of SDBS is too high, the surface of carbon nanotubes may form an excessively thick layer of negative charges. This thick layer of charge can create a shielding effect between carbon nanotubes, reducing effective contact between them and thus impeding the formation of conductive networks.

**Figure 7:**
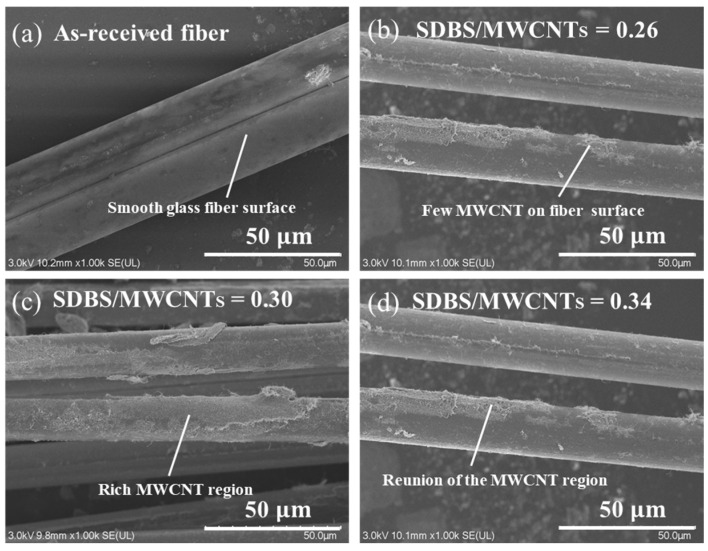
SEM micrographs of MWCNT-GF after impregnation in solution with different dispersant concentrations: (**a**) surface of as-received fibers, (**b**) SDBS/MWCNT_S_ = 0.26, (**c**) SDBS/MWCNT_S_ = 0.30, and (**d**) SDBS/MWCNT_S_ = 0.34.

Figure 7 shows SEM micrographs of MWCNT-coated glass fibers after impregnation in solutions with different dispersant concentrations. Figure 7a shows the surface of the as-received glass fiber without impregnation, which is smooth and not covered with carbon nanotubes. Figure 7b shows the glass fiber surface at an SDBS/MWCNT ratio of 0.26, showing a small but unevenly distributed number of carbon nanotubes. At the SDBS/MWCNT ratio of 0.3, the degree of carbon nanotube coverage increases, and the distribution is relatively uniform. However, for the higher SDBS/MWCNT ratio of 0.34, carbon nanotubes begin to aggregate, thus affecting the uniformity of the conductive network. Therefore, a SDBS/MWCNT ratio of 0.3 was selected to prepare carbon nanotube impregnation solution.

**Figure 8:**
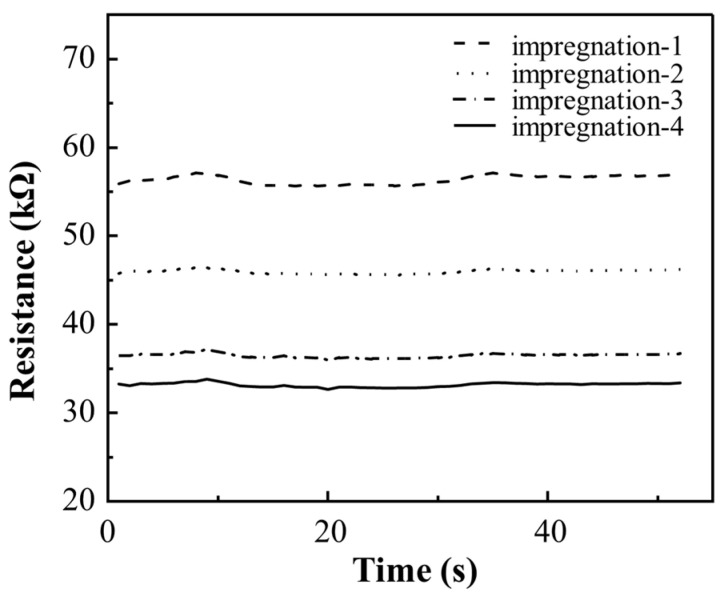
The change in resistance of MWCNT-GF after different cycles of impregnation.

The permeability threshold of the MWCNTs on glass fibers also depends on the number of cycles of impregnation. Figure 8 shows the change in resistance of MWCNT-coated glass fibers after different cycles of impregnation. It can be seen from Figure 8 that with the increase in impregnation time, the resistance value of the fiber gradually decreases and tends to be stable. At the first impregnation, the average resistance value is 56.7 kΩ, which indicates that the carbon nanotubes have not formed a complete conductive network, resulting in high resistance. After two cycles of impregnation, the resistance value decreased significantly to about 46.1 kΩ, and the coverage and distribution uniformity of carbon nanotubes were improved, and the conductive network gradually formed. After three cycles of impregnation, the resistance value is further reduced to about 36.6 kΩ, indicating that the carbon nanotubes coating is more uniform, and the conductive network is basically realized. The resistance value tended to stabilize without significant change and remained at about 33.3 kΩ for the four cycles of impregnation, demonstrating that the carbon nanotubes on the glass fiber surface have reached the permeability threshold, and the effect of resistance reduction is very limited after repeated impregnation.

**Figure 9:**
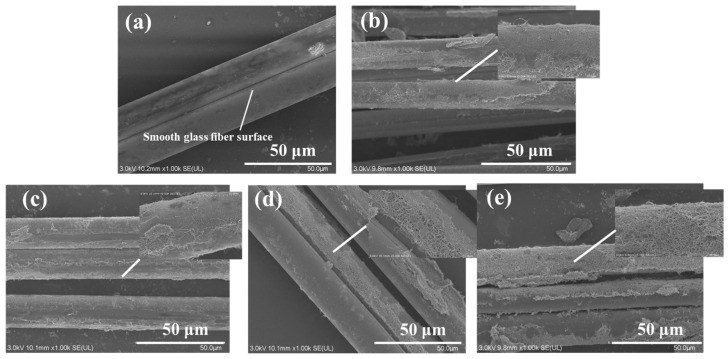
SEM micrographs of MWCNT-GF after different cycles of impregnation: (**a**) surface of as-received fibers, (**b**) impregnation for 1 cycle, (**c**) impregnation for 2 cycles, (**d**) impregnation for 3 cycles, and (**e**) impregnation for 4 cycles.

**Figure 10:**
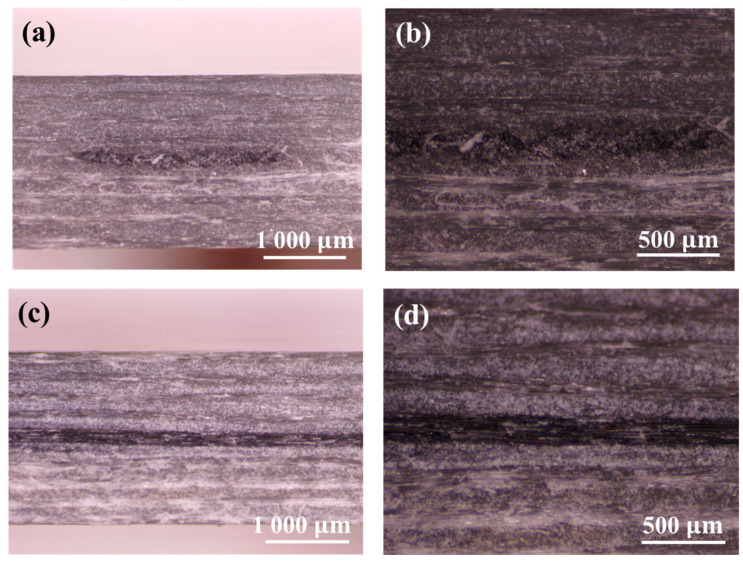
Microscope observation of cross-section of laminates with sensing fibers before impact: (**a**) warp sensing fiber at magnification of 105×, (**b**) warp sensing fiber at magnification of 210×, (**c**) weft sensing fiber at magnification of 105×, and (**d**) weft sensing fiber at magnification of 210×.

SEM micrographs of MWCNT-coated glass fibers after different impregnation cycles are given in Figure 9. By comparing the surface morphology of fibers after different impregnation cycles, the variation in the coating degree and uniformity of carbon nanotubes on the glass fiber surface could be clearly observed. Figure 9a–c show the as-received glass fiber surface and the glass fiber surface after one and two impregnations, respectively. It could be observed that the coating of carbon nanotubes increases significantly, and the distribution becomes more uniform, but there are still a few incomplete areas. When the glass fiber was immersed three times, the carbon nanotubes formed a uniform and continuous coating on the fiber surface, almost completely covering the fiber surface, forming a relatively ideal conductive network structure. Based on SEM images and resistance test results, different impregnation cycles have a significant effect on the conductivity of MWCNT-GF. With the increase in impregnation time, the coating degree and uniformity of carbon nanotubes on the surface of glass fiber are gradually improved, as well as the conductive network, which significantly reduces the resistance value of the MWCNT-GF. After the third impregnation cycle, the MWCNT coating basically forms a uniform and continuous conductive network, the resistance value tends to be stable, and the further increase in impregnation time has little effect on the improvement in conductivity. Therefore, in practical applications, the three cycles of impregnation can ensure the excellent electrical conductivity and stable sensing capability of MWCNT-GF. Figure 10 illustrates the microscope observation results of cross-sections of laminates with sensing fibers before impact. It could be confirmed that the interface between the sensing fibers and the adjacent layer of the laminate is well combined in terms of both the warp and the weft of the sensing fibers, which is also verified by the following results of the low-velocity impact tests.

### 3.2. Low-Velocity Impact Test Results

**Figure 11:**
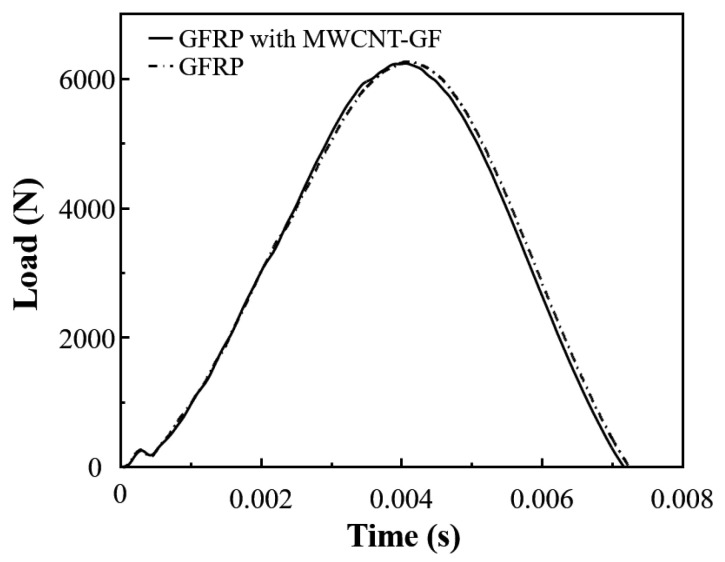
Typical load–time curves of composite laminates with and without the MWCNT-GF under low-velocity impact of 20 J.

**Figure 12:**
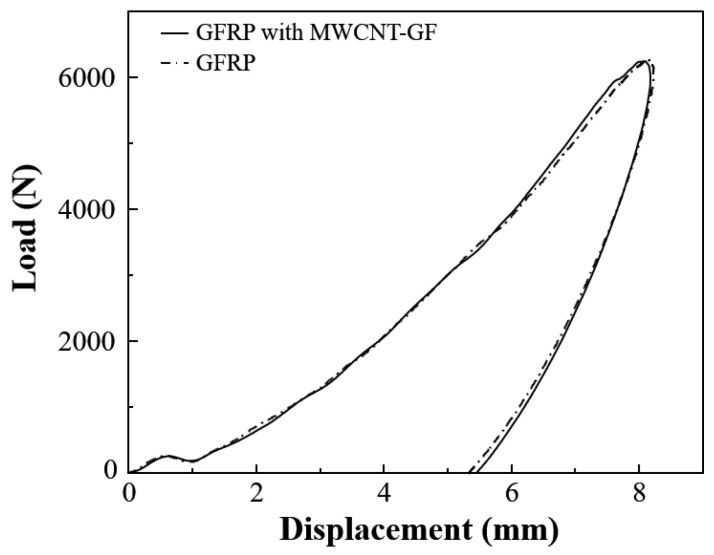
Typical load–displacement curves of composite laminates with and without the MWCNT-GF under low-velocity impact of 20 J.

**Figure 13:**
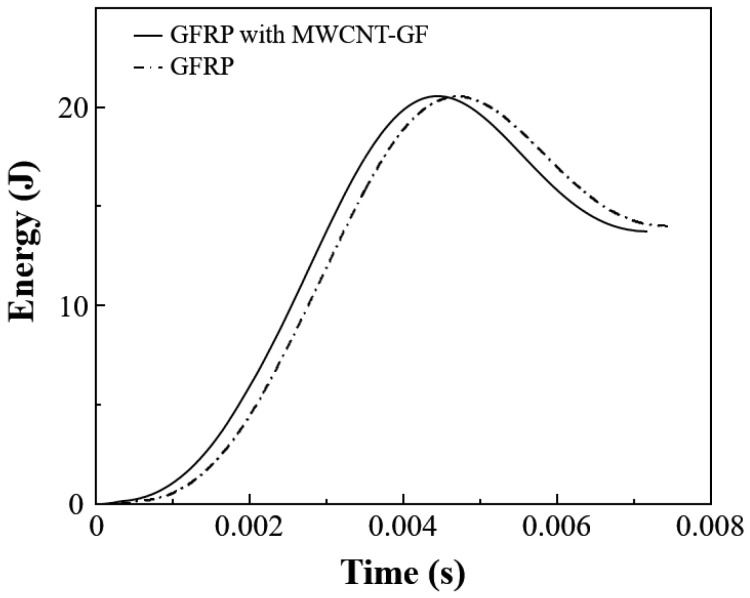
Typical energy–time curves of composite laminates with and without the MWCNT-GF under low-velocity impact of 20 J.

**Figure 14:**
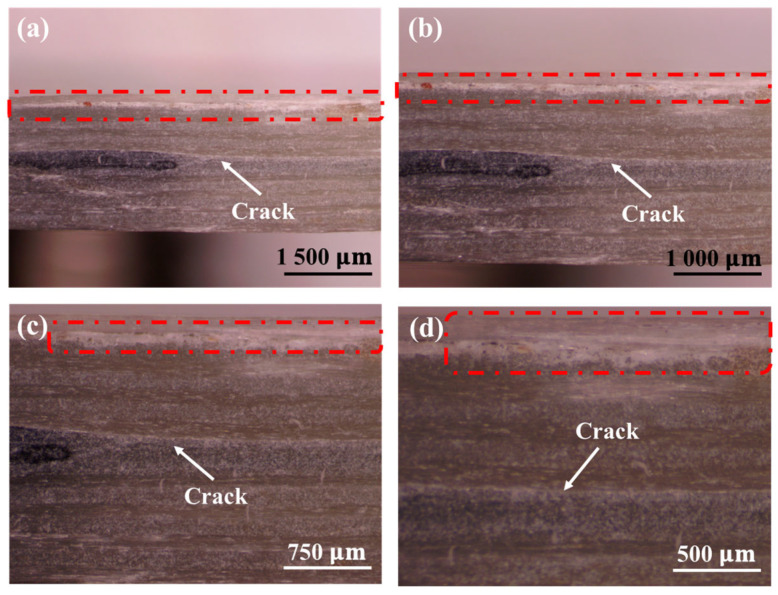
Microscope observation of cross-section of laminates with sensing fibers after impact: (**a**) magnification of 70×, (**b**) magnification of 105×, (**c**) magnification of 140×, (**d**) magnification of 210×.

The traditional rigid embedded sensors usually have a negative effect on the impact resistance of the composite laminates. Therefore, it is particularly important to carry out low-velocity impact tests on the composite laminates with and without the MWCNT-GF sensing layer. The drop weight impact testing machine accurately provides the load–time curve of the drop hammer via the force sensor, which is used to calculate the acceleration change for the drop hammer. Then, the load–displacement and energy–time curves are obtained through the integral calculation of the acceleration of the drop hammer. Due to the good repeatability of the low-velocity impact curves, the typical test curves are selected for analysis for the convenience of clear comparison. As shown in Figure 11, the load–time curves of GFRP laminates with and without an MWCNT-GF sensing layer show high consistency under a low-velocity impact of 20 J, indicating that the embedment of the MWCNT-GF sensing layer has no significant effect on the impact response of the laminates. The load–displacement curve can reflect the deformation behavior of materials during impact. As shown in Figure 12, the load–displacement curves of GFRP laminates with and without the MWCNT-GF sensing layer are also highly similar. In the low-velocity impact test, the embedment of the MWCNT-GF sensing layer does not change the maximum load and the corresponding displacement of laminates, indicating that it has little influence on the overall deformation characteristics of laminates. Energy–time curves are used to assess the energy absorption capacity of the material during the impact process. As shown in Figure 13, the energy–time curves of GFRP laminates with and without the MWCNT-GF sensing layer are almost the same. However, the energy absorption of GFRP laminates containing MWCNT-GF occurs about 10% earlier than that of GFRP laminates overall. The statistical results of low-velocity impact tests of laminates with and without sensing fibers, including load, displacement, and absorbed energy, are listed in Table 2. As seen from the microscope observation of cross-sections of laminates with sensing fibers after impact shown in Figure 14, the impact side of the laminate has obvious damage under low-velocity impact, including delamination and matrix damage, as marked by the red dotted line box. Meanwhile, fine delamination cracks also appeared along both sides of the sensing fibers, while no similar cracks were observed between the other adjacent glass fiber layer, which should be responsible for the about 10% advance in the energy absorption curve of the GFRP composite with MWCNT-GF. It is indicated that since the sensing fiber bundles are distributed in the form of an array, there is interface weakening between adjacent glass fiber layers. Therefore, damage will occur earlier under the impact load, so as to absorb energy. Because this part of the interface damage is very tiny, it has no impact on the overall energy absorption performance of the laminates.

### 3.3. Damage Monitoring of MWCNT-GF

**Figure 15:**
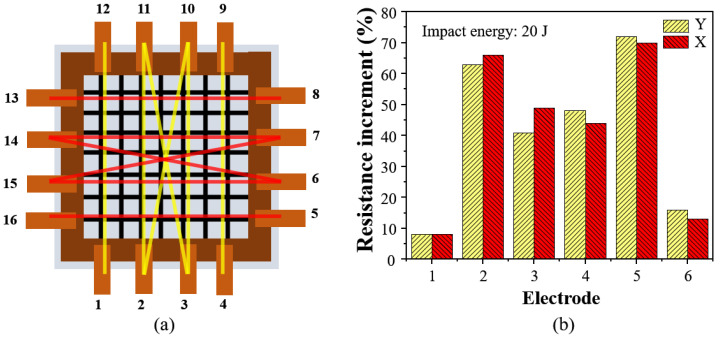
(**a**) Coding method of the sensor, and (**b**) percentage increase in resistance between electrodes in the sensing layer under a low-velocity impact of 20 J.

In order to systematically evaluate the performance and stability of the MWCNT-GF sensing layer, the composite laminate electrodes embedded in the sensing layer are labeled and measured, as shown in Figure 15a. The damage of the impact region decreased the conductivity of the sensing layer, and the difference in the resistance of sensing fibers is measured before and after the impact to analyze the area of the damage region using ERT technology. The electrode pairs selected in the X direction are numbered sequentially as 8–13, 14–6, 14–7, 15–7, 15–6, and 16–5, while, 12–1, 11–3, 11–2, 10–3, 10–2, and 9–4 are the corresponding electrode pairs in the Y direction. Figure 15b shows the percentage increase in relative resistance of each electrode pair after low-velocity impact. Because of the relatively low impact energy applied in this study, the matrix and delamination damage occur inside the composite laminates under low-velocity impact, which are detected via the MWCNT-GF sensing layer. Especially, the sensing layer located at the impact point exhibits the most serious damage due to the direct impact. Therefore, the resistance changes for the 2–10, 3–11, 6–14, and 7–15 electrode pairs are the largest, reaching 60%-70%. In addition, because the composite laminate is fixed in the fixture, the resistance changes for the 1–12, 4–9, 5–16, 8–13 electrode pairs also respond to the impact, but the amplitude is small. It could be found from the experimental results that the MWCNT-GF sensing layer has a high sensitivity to the damage in the composite laminate, especially the damage response to the direct impact position, which indicates that it has potential application significance in structural health monitoring of composite laminates.

**Figure 16:**
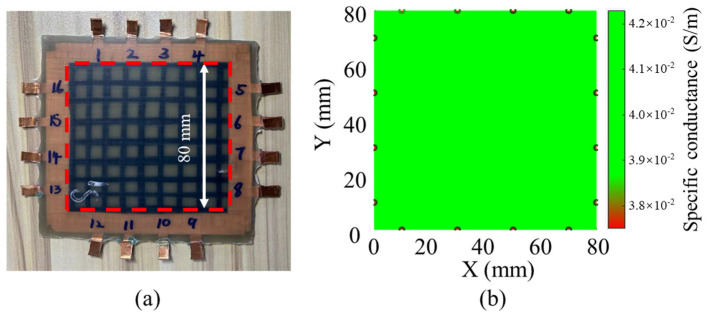
(**a**) Specimen surface before impact, and (**b**) ERT image after normalization of sensing layer before impact.

**Figure 17:**
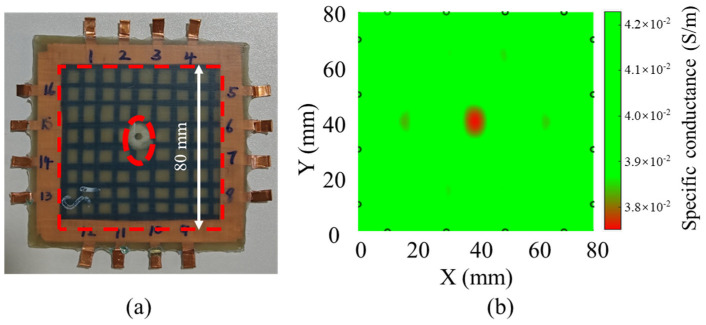
(**a**) Specimen surface after low-velocity impact of 20 J, and (**b**) ERT image after normalization of sensing layer after low-velocity impact of 20 J.

Figure 16 and Figure 17 show the ERT imaging results of the specimens before and after a low-velocity impact of 20 J, as well as the corresponding morphology of the impact side of the specimens. In this study, the display threshold is set according to the range of the measured resistance to improve the display differentiation. As shown in Figure 16, the imaging results before the impact show uniform resistance of about 4.1 × 10^−2^ S/m in the entire region without obvious abnormality, which is consistent with the expectation of a uniform film layer after normalization. After the low-velocity impact of 20 J, the imaging results changed significantly. As shown in Figure 17, the impact area showed the obvious difference in conductance values below 3.8 × 10^−2^ S/m, which decreased by more than 7.3%, reflecting the local resistance change caused by impact damage. In addition, several small regions of high resistance also appeared in the image. Although these regions are not the main damage areas, they may reflect the secondary damage caused by the sensing layer during the impact process, such as local stress concentration or change in the internal microstructure of sensing fibers. Since GFRP laminates have good light transmission, the internal damage of laminates can be observed simply via irradiation with a strong light source, and the damage area could be conveniently measured and calculated. By comparing the imaging results before and after impact, the effectiveness of ERT technology in detecting and locating internal damage in composite laminates can be clearly confirmed. To evaluate the accuracy of ERT imaging, the damage area specimen after impact and ERT imaging results are compared, as shown in Table 3. The result shows that the actual damage area of the specimen is close to that of ERT imaging, with an error of only 4.5%, which indicates the good accuracy of the damage monitoring of the composite laminates via ERT imaging technology.

## 4. Conclusions

In this study, MWCNT-modified glass fibers are embedded into composite laminates as a sensing layer, and normalized ERT technology is used to monitor the damage of laminates after low-velocity impact. The following conclusions are drawn.

(1) The specific effects of different impregnation schemes on the electrical properties of MWCNT-GF are investigated. When the carbon nanotube concentration reaches 6.25 mg/mL, a stable and effective conductive network forms on the surface of glass fibers. The dispersion of carbon nanotubes is better at the right SDBS/MWCNT ratio of 0.3. The carbon nanotubes on the glass fiber surface have basically reached the permeability threshold after three cycles of impregnation;

(2) Low-velocity impact tests demonstrate that the embedment of an MWCNT-GF sensing layer has no significant effect on the impact response of the laminates, although the energy absorption of GFRP laminates containing MWCNT-GF occurs 10% earlier than that of GFRP laminates overall;

(3) The resistance changes greatly before and after damage, and the corresponding electrode pair resistance changes near the impact point are about 60–70%. The actual damage area of the specimen is close to that of ERT imaging, with an error of only 4.5%, indicating the good accuracy of the damage monitoring of the composite laminates via ERT imaging technology.

## Figures and Tables

**Table 1:** Preparation parameters for MWCNT-modified glass fibers.

Specimen Number	MWCNT (mg)	SDBS (mg)	Deionized Water (mL)	Number of Impregnation Cycles
A	75	22.5	20	1
B	100	30	20	1
C	125	37.5	20	1
D	150	45	20	1
E	125	32.5	20	1
F	125	42.5	20	1
G	125	37.5	20	2
H	125	37.5	20	3
I	125	37.5	20	4

**Table 2:** Statistical results of low-velocity impact tests of laminates with and without sensing fibers.

Specimen	Average Peak Force (kN)/(SD)	Average Maximum Displacement (mm)/(SD)	Average Absorbed Energy (J)/(SD)
GFRP	6.199 (0.071)	8.224 (0.019)	14.008 (0.255)
GFRP with MWCNT-GF	6.289 (0.046)	8.207 (0.018)	14.038 (0.218)

**Table 3:** Comparison of the actual damage area of the specimen after low-velocity impact and ERT measuring results.

Average Actual Damage Area (mm^2^)/(SD)	ERT Measuring Area (mm^2^)/(SD)	Error (%)
146.135 (6.745)	139.49 (5.843)	4.55

## Data Availability

The datasets analyzed during the current study are not publicly available due to the nature of this research but are available from the corresponding author on reasonable request.
